# Blast-Induced Tinnitus and Elevated Central Auditory and Limbic Activity in Rats: A Manganese-Enhanced MRI and Behavioral Study

**DOI:** 10.1038/s41598-017-04941-w

**Published:** 2017-07-07

**Authors:** Jessica Ouyang, Edward Pace, Laura Lepczyk, Michael Kaufman, Jessica Zhang, Shane A. Perrine, Jinsheng Zhang

**Affiliations:** 1 Department of Otolaryngology and Head and Neck Surgery, Wayne State University School of Medicine, Detroit, MI 48201 USA; 2 Department of Psychiatry and Behavioral Neurosciences, Wayne State University School of Medicine, Detroit, MI 48201 USA; 3 Department of Communication Sciences & Disorders, Wayne State University College of Liberal Arts and Sciences, Detroit, MI 48201 USA

## Abstract

Blast-induced tinitus is the number one service-connected disability that currently affects military personnel and veterans. To elucidate its underlying mechanisms, we subjected 13 Sprague Dawley adult rats to unilateral 14 psi blast exposure to induce tinnitus and measured auditory and limbic brain activity using manganese-enhanced MRI (MEMRI). Tinnitus was evaluated with a gap detection acoustic startle reflex paradigm, while hearing status was assessed with prepulse inhibition (PPI) and auditory brainstem responses (﻿ABR﻿﻿s). Both anxiety and cognitive functioning were assessed using elevated plus maze and Morris water maze, respectively. Five weeks after blast exposure, 8 of the 13 blasted rats exhibited chronic tinnitus. While acoustic PPI remained intact and ABR thresholds recovered, the ABR wave P1-N1 amplitude reduction persisted in all blast-exposed rats. No differences in spatial cognition were observed, but blasted rats as a whole exhibited increased anxiety. MEMRI data revealed a bilateral increase in activity along the auditory pathway and in certain limbic regions of rats with tinnitus compared to age-matched controls. Taken together, our data suggest that while blast-induced tinnitus may play a role in auditory and limbic hyperactivity, the non-auditory effects of blast and potential traumatic brain injury may also exert an effect.

## Introduction

Subjective tinnitus is phantom auditory perception that occurs without an external source. It can be very distressing and is associated with anxiety, annoyance, irritability, disturbed sleep patterns, and depression^1–5^. The most common cause of tinnitus is acoustic trauma, which can range from occupational noise to blast overpressure exposure. Due to the prominent usage of improvised explosive devices and rocket-assisted mortars in modern war theaters, blast-induced tinnitus has become an increasingly significant health problem for military personnel and veterans^6, 7^. Indeed, it has been reported that 49% of blast-injured service members report tinnitus perception and 60% sustain hearing loss^8^. Individuals with blast-related traumatic brain injury (TBI), the most common injury type incurred in the aforementioned conflicts^9^, presented with the highest rates of tinnitus and hearing loss. Furthermore, tinnitus was the number one service-connected disability affecting military personnel, resulting in nearly two billion dollars in annual disability compensation^10^. Despite the adverse health and economic consequences of tinnitus, however, its underlying neuropathophysiology are not fully understood, which contributes to the lack of reliable treatment strategies.

Available evidence indicates that maladaptive changes in the central auditory system^11, 12^, as well as non-auditory centers like the limbic system (see ref. 13 for review), are correlated with tinnitus perception and tinnitus-like behavior in humans and animals, respectively^14^. In the central auditory system, neuroimaging studies in tinnitus patients have shown abnormal sound-evoked activity in the cochlear nuclei and inferior colliculi^15–19^, as well as abnormal brain metabolism^20–26^, and alterations in microstructural integrity^27–32^ and gray matter volume^33–35^. Non-auditory brain regions like the limbic system are also targets for study, given that limbic-associated functioning, including cognitive, emotional, and psychological well-being, are frequently compromised in tinnitus sufferers^36–44^. Tinnitus patients have shown increased cerebral blood flow in the hippocampus, amygdala, and anterior cingulate cortex^24, 25, 45^, hyperactivity in the nucleus accumbens^46^, increased connectivity between the parahippocampus and auditory resting state network^47^, and decreased grey matter in the hippocampus^33^ and nucleus accumbens^48^. The neuropathophysiology of tinnitus resulting from blast trauma, however, has not been as well-studied as other etiologies and requires further investigation.

To date, some detailed effects of blast on the auditory system have been documented, such as outer hair cell and spiral ganglion loss^49^, expression of deafness genes^50^ and biomarkers for astrocytosis and axonal injury, among others^51^ These effects, however, may not be tinnitus-specific. Recently, our lab found evidence suggesting structural damage and compensatory structural changes in the inferior colliculus (IC) and medial geniculate body (MGB) of rats with blast-induced tinnitus^52^, in addition to acute and chronic hyperactivity or hypoactivity in the dorsal cochlear nucleus (DCN)^53^ IC^54^, and auditory cortex (AC)^55^. Notably, some incongruities between blast and noise-induced tinnitus are apparent, such as but not limited to altered neural activity occurring in different frequency regions and time points^53–55^.

One promising tool that can be used to study tinnitus is manganese-enhanced magnetic resonance imaging (MEMRI)^56^. MEMRI uses manganese as an activity-dependent paramagnetic contrast agent that accumulates in active neurons through voltage-gated calcium channels^57, 58^. The key advantages of MEMRI are that it measures slow efflux of manganese from cells, enables assessment of animals while they are conscious, and possesses higher spatial resolution than fMRI. More importantly, since manganese accumulation occurs prior to MRI scanning, the collected data is not contaminated by loud noise generated by the MRI scanner. Thus far, MEMRI studies on animals with tinnitus have found increased manganese accumulation, or activity, in the DCN, ventral cochlear nucleus (VCN), IC, and paraflocculus^59–62^.

In the current study, we subjected rats to unilateral 14 psi blast exposure and assessed them for tinnitus using a gap-detection acoustic startle reflex paradigm (GAP) and for hearing loss using prepulse inhibition (PPI) and auditory brainstem responses (ABRs). Five weeks following blast exposure, we performed Morris water maze (MWM) and elevated plus maze (EPM) testing to assess spatial cognition and anxiety, respectively. This was followed by MEMRI to study the effect of blast-induced tinnitus and its associated TBI on activation of the central auditory and limbic systems. Our results showed that that blast exposure significantly elevated manganese accumulation in rats with tinnitus behavior in a bilateral fashion compared to control rats, although manganese accumulation was not greater in tinnitus(+) versus tinnitus(−) rats. Our data suggests that blast-induced tinnitus may play a role in brain activation, but the non-auditory effects of blast and potentially-induced TBI must also be considered.

## Results

### Gap-detection and prepulse inhibition (PPI) acoustic startle reflex testing

**Figure 1:**
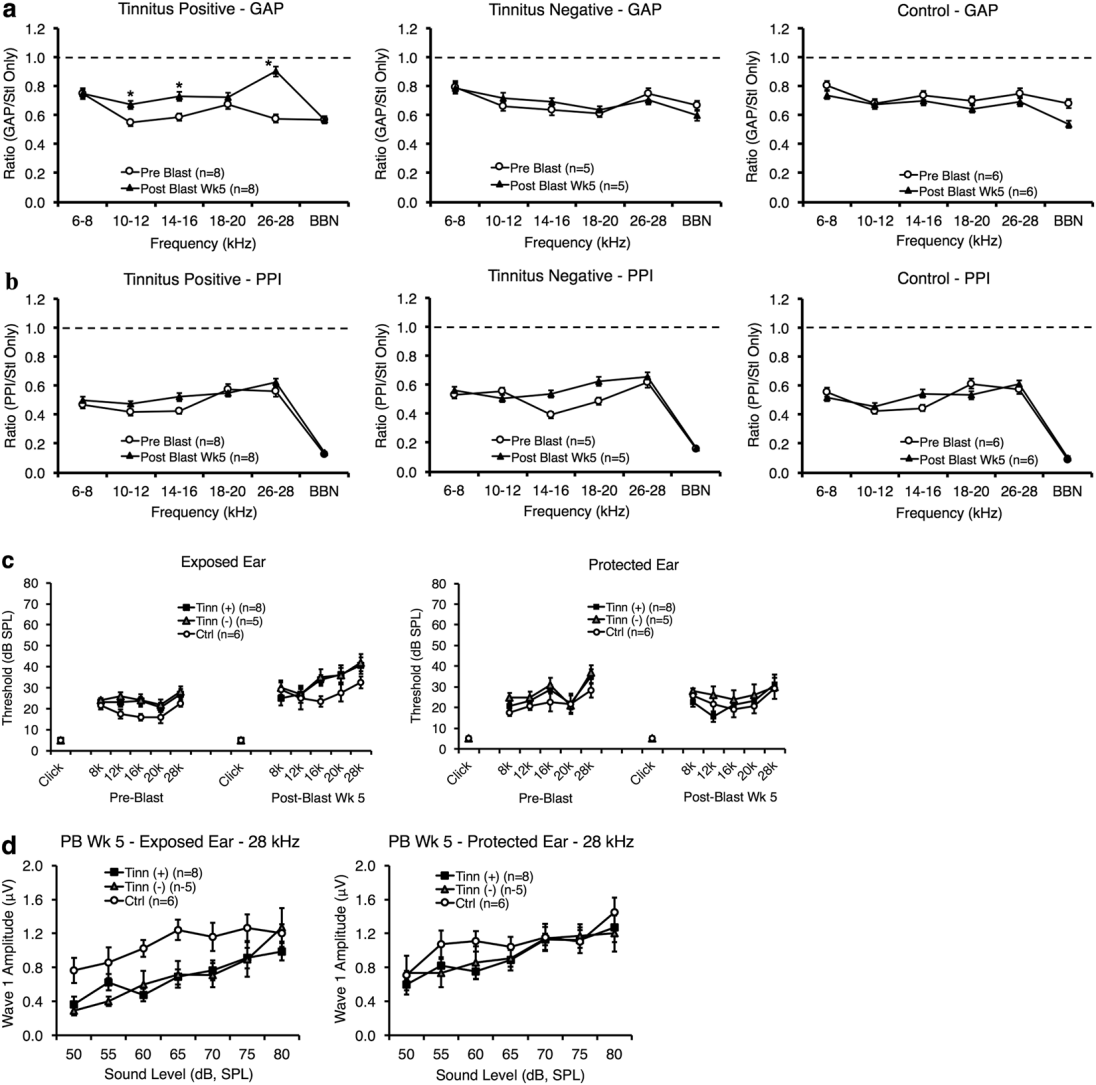
Some blast-exposed rats exhibit tinnitus behavior, while there were no observed differences in hearing impairment between tinnitus(+) and tinnitus(−) rats. (**a**,**b**) Gap-detection (**a**) and PPI ratio values (**b**) are shown for both pre-blast and post-blast week 5 for the tinnitus(+), tinnitus(−), and control (ctrl) groups. Tinnitus(+) rats exhibited moderate tinnitus between 10–16 kHz and robust tinnitus at 26–28 kHz. No significant increases in post-blast PPI ratio values were seen for any group. The dotted line indicates a ratio value of 1, or maximum tinnitus (GAP) or auditory detection deficits (PPI). (**c**) ABR thresholds were measured in response to click and tone-burst stimuli before blast exposure and at 5 weeks after blast exposure. Despite some moderate differences, there were no significant threshold differences between any group for any ear, time point, or frequency. (**d**) ABR wave P1-N1 amplitudes were plotted as a function of sound intensity in blast-exposed and plug-protected ears for the tinnitus(+), tinnitus(−), and control groups. Amplitude reductions in blast-exposed ears were observed across most intensities in tinnitus(+) and tinnitus(−) rats compared to controls. No significant differences, however, were found in plug-protected ears. For all graphs, error bars represent the standard error of the mean. * indicates specific significance (p < 0.05).

Of the thirteen blast-exposed rats, eight exhibited post-blast gap-detection ratios that were significantly higher than pre-blast ratios and that were not significantly lower than post-blast startle only ratios, thus indicating tinnitus-like behavior. These gap-detection deficits occurred through five weeks post-blast at a frequency range of 10–28 kHz, although the 26–28 kHz region was the most common and robust frequency band among individual animals. On a group level, repeated measures ANOVA showed that tinnitus(+) rats exhibited a significant interaction between time and frequency (*F*_(5,27)_ = 5.798, *p* < 0.001). Post-hoc t-tests indicated significant elevation in the post-blast gap-detection ratios at 10–12 (*t*[31] = 3.548 *p* = 0.001), 14–16 (*t*[31] = 2.377 *p* = 0.024), and especially at 26–28 kHz (*t*[31] = 5.489 *p* < 0.001; Fig. 1a). For PPI ratio data (Fig. 1b), repeated measures ANOVA showed no significant effect of time (*F*_(1,31)_ = 1.833, *p* = 0.186) or interaction between time and frequency (*F*_(5,27)_ = 0.931, *p* = 0.477), indicating that auditory detection was not impaired and thus did not confound gap-detection deficits. Both the tinnitus(−) and control group showed no significant effects for time or time and frequency interactions in gap-detection or PPI ratio data, although an insignificant increase in post-blast tinnitus(−) PPI ratios was observed at 14–16 and 18–20 kHz. This suggests an absence of tinnitus perception and auditory detection deficits in the tinnitus(−) and control groups.

To determine whether there were overall changes in startle amplitude and whether these changes could confound detection of tinnitus behavior^63^, we also plotted startle amplitude changes in individual animals (see Supplementary Fig. S1). We found that, relative to baseline startle amplitude, post-blast startle amplitude either increased, stayed the same, or decrease in individual animals. However, these changes did not primarily occur in the tinnitus positive group. Therefore, it did not appear that overall changes in startle amplitude, such as floor effects, confounded tinnitus positive behavior.

In addition, we compared gap-detection and PPI ratios of rats blasted under ketamine/xylazine anesthesia with those of rats blasted under isoflurane (data not shown). Repeated measures ANOVA showed no group differences in post-blast gap-detection ratios (*F*_(1,50)_ = 0.146, *p* = 0.704) or PPI ratios (*F*_(1,50)_ = 0.049, *p* = 0.826), indicating that the anesthesia adopted did not have a significant effect on blast-induced tinnitus or auditory detection. Of the 6 rats blasted under ketamine/xylazine, 3 exhibited tinnitus at 5 weeks post-blast while 3 rats did not. Of the 7 rats blasted under isoflurane, 5 exhibited tinnitus at 5 weeks post-blast while 2 rats did not.

### Auditory brainstem responses (ABRs)

ABR thresholds were measured before and five weeks after blast exposure in the left ear (blasted) and right ear (plug-protected) to determine if there were lasting hearing threshold shifts following blast. Repeated measures ANOVA showed no significant interactions between time, frequency and group for the left ear (*F*_(10,26)_ = 1.278, *p* = 0.293; Fig. 1c) or the right ear (*F*_(10,26)_ = 1.673, *p* = 0.141; Fig. 1c). There were also no interactions between group and time (left ear *F*_(2,16)_ = 0.003, *p* = 0.997; right ear *F*_(2,16)_ = 0.375, *p* = 0.693) or differences between groups (left ear *F*_(2,16)_ = 2.822, *p* = 0.089; right ear *F*_(2,16)_ = 1.049, *p* = 0.373). Thus, while small threshold shifts were noticeable in both tinnitus( + ) and tinnitus(−) rats, these shifts did not reach statistical significance. When we compared the hearing thresholds of rats blasted under ketamine/xylazine with those blasted under isoflurane, repeated measures ANOVA showed no significant interactions between time, frequency, and group for the left ear (*F*_(5,7)_ = 0.505, *p* = 0.765), or the right ear (*F*_(3.196,5)_ = 0.412, *p* = 0.757), or any other interactions or differences between groups.

Since temporary threshold-shifts or recovered hearing thresholds often represents hidden hearing loss and are accompanied by ribbon synapse losses and degraded amplitude of P1-N1^64^, we examined wave P1-N1 amplitudes at 5 weeks post-blast to assess the cochlear condition. ANOVA revealed a significant overall reduction in P1-N1 amplitude at 28 kHz in the left ear (*F*_(2,118)_ = 12.067, *p* < 0.001; Fig. 1d) for both tinnitus(+) and tinnitus(−) animals, compared to controls. No significant amplitude reductions, however, were observed in the right ear (*F*_(2,121)_ = 1.649, *p* = 0.197; Fig. 1d), suggesting unilateral cochlear impact. When we compared the P1-N1 amplitudes of rats blasted under ketamine/xylazine with those blasted under isoflurane (see Supplementary Fig. S2), we also found no significant differences at 28 kHz in the left ear (*F*_(2,79)_ = 0.288, *p* = 0.593) or in the right ear (*F*_(2,81)_ = 0.006, *p* = 0.939).

### Elevated plus maze (EPM)

**Figure 2:**
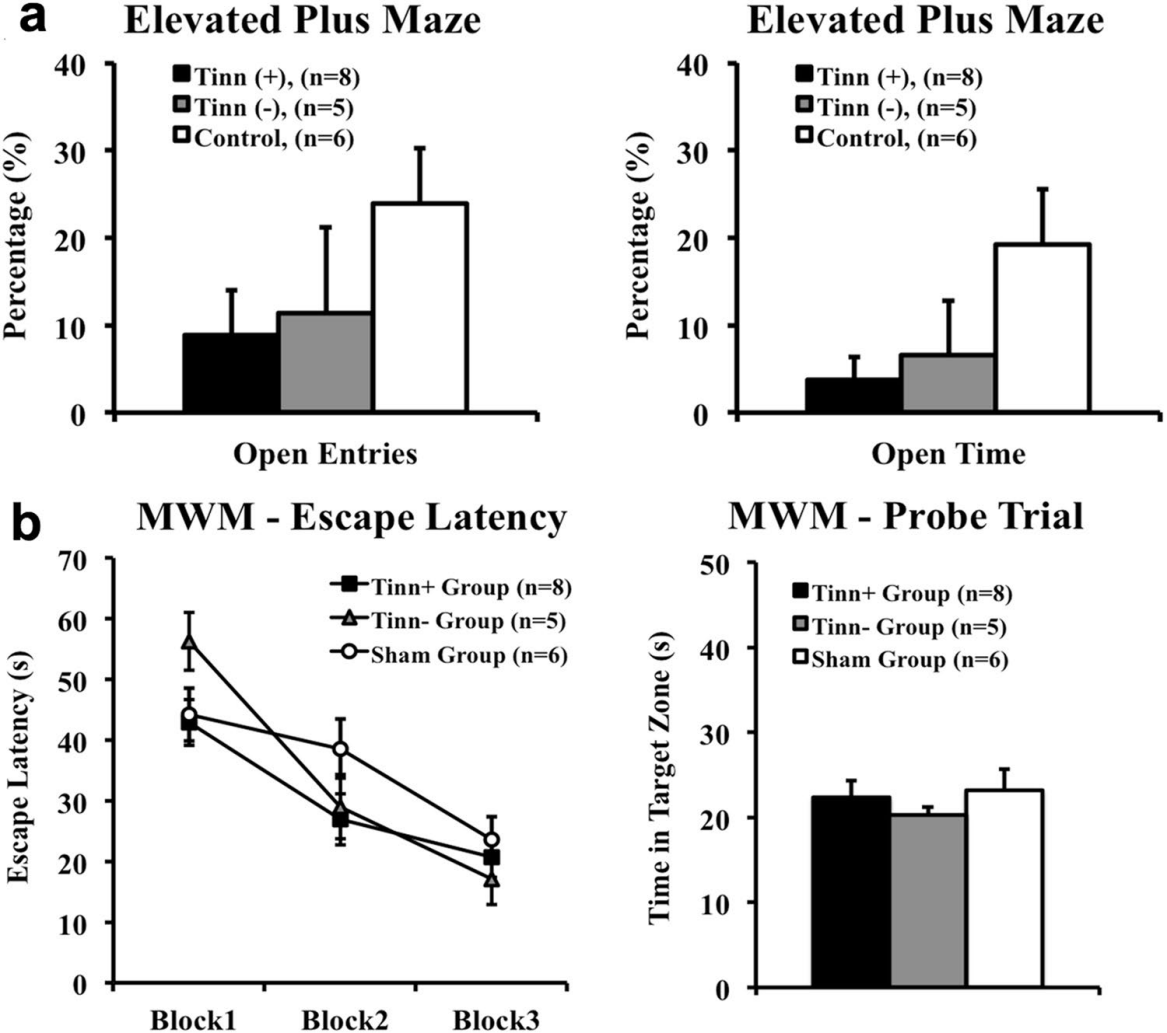
Blast-exposed rats as a whole exhibited significant anxiety, but no significant deficits in spatial learning or memory. (**a**) On the elevated plus maze (EPM), the only significant between-group difference was how blasted rats as a whole (tinnitus(+) and tinnitus(−) rats) committed significantly less open-arm time compared to control (ctrl) rats. Rats were tested five weeks following blast and were compared based on the percent of open-arm entries and time, relative to total entries and time. (**b**) No significant between-group differences were observed during escape latency trials or the probe trial of the Morris water maze (MWM). Rats were tested five weeks following blast. Each block consisted of 4 escape latency trials, where latency to locate the hidden platform was measured. Time spent in the former hidden platform quadrant, or target zone, was measured in the probe trial. For all graphs, error bars represent standard error of the mean. *Indicates significance (p < 0.05) between blasted rats as a whole (tinnitus(+) and tinnitus(−) rats) vs. control rats.

Rats were tested on the EPM to assess the impact of blast and tinnitus on anxiety-like behavior. ANOVA revealed no significant differences in total-arm entries between any of the three groups (*F*_(2.16)_ = 2.801, *p* > 0.091; Table 1), indicating that there were no differences in mobility between groups. While the tinnitus(+) and tinnitus(−) groups spent less time in the open arms and committed less open-arm entries compared to the control group, there were also no significant differences between any of the three groups (*F*_(2.16)_ = 2.925, *p* > 0.083; Fig. 2a). Only when tinnitus(+) and tinnitus(−) groups were combined did they demonstrate significantly less time spent in the open-arms, compared to controls (*t*[17] = 2.443 *p* = 0.026; Fig. 2a). This suggests that the effects of blast exposure, regardless of tinnitus presence, elevated anxiety levels for at least five weeks.

**Table 1:** Number of open- and closed-arm, and total arm entries for the tinnitus(+), tinnitus(−), and control groups. Data are presented as mean ± standard error of the mean. Notably, the number of total-arm entries was not significantly lower in tinnitus(+) or tinnitus(−) rats compared to controls, indicating that mobility was not impaired by blast exposure.

Epm: Number of Entries			
Group	Open Arm	Closed Arm	Total
Tinnitus (+), (n = 8)	0.75 ± 0.42	7.25 ± 0.67	8.50 ± 0.82
Tinnitus (−), (n = 5)	1.20 ± 0.97	6.80 ± 1.83	8.00 ± 1.94
Control, (n = 6)	2.83 ± 0.82	7.75 ± 0.85	10.58 ± 1.07

### Morris water maze (MWM)

#### Escape latency trials

To assess spatial learning, rats underwent 12 escape latency trials (3 blocks, 4 trials per block) to gauge the effect of blast exposure and tinnitus on the time needed to find a hidden platform. Repeated measures ANOVA showed that there was no significant difference between groups (*F*_(2,16)_ = 1.094, *p* = 0.359) and no significant interaction between block and group (*F*_(4,32)_ = 1.977, *p* = 0.122; Fig. 2b). Therefore, it appeared that the currently used single blast exposure did not significantly affect their spatial learning under these conditions.

#### Probe trial

Following the third block of the spatial acquisition task, rats were tested using a probe trial to assess spatial memory. Similar to spatial learning, repeated measures ANOVA revealed no significant differences between groups on time spent in the target zone (*F*_(2,16)_ = 1.302, *p* = 0.299), suggesting that the single blast exposure did not affect spatial memory under these conditions (Fig. 2b).

### Manganese-enhanced MRI (MEMRI) – auditory structures

MEMRI was conducted to determine whether blasted rats with or without tinnitus experienced brain activity changes in the central auditory system. We found an overall group effect (*F*_(2,16)_ = 6.312, *p* = 0.010) when comparing the three groups, with the tinnitus(+) group specifically exhibiting higher contrast-to-noise ratios (CNRs), or manganese accumulation, in auditory structures compared to the control group (*p* = 0.008). Repeated measures ANOVA showed no interactions between group and hemisphere or brain region, and Bonferroni adjustments showed no overall group differences in auditory structures between the tinnitus(+) and tinnitus(−) group (*p* = 0.435), or between the tinnitus(−) and control group (*p* = 0.311). Although some differences in manganese accumulation occurred between left and right side structures of the control group, which may be partially due to small sample size (n = 5), there were no statistically significant interactions between hemisphere (*F*_(1,1)_ = 2.065, *p* = 0.210) or between hemisphere and brain region (*F*_(2.406,6)_ = 0.701, *p* = 0.540) within the control group. There were also no overall group differences in manganese uptake between noise (*F*_(2,16)_ = 0.824, *p* = 0.456) and the averaged anterior pituitary (*F*_(2,16)_ = 0.414, *p* = 0.668). The latter validates our normalization method and indicates that there were no systemic, non-specific enhancements in manganese uptake.

**Figure 3:**
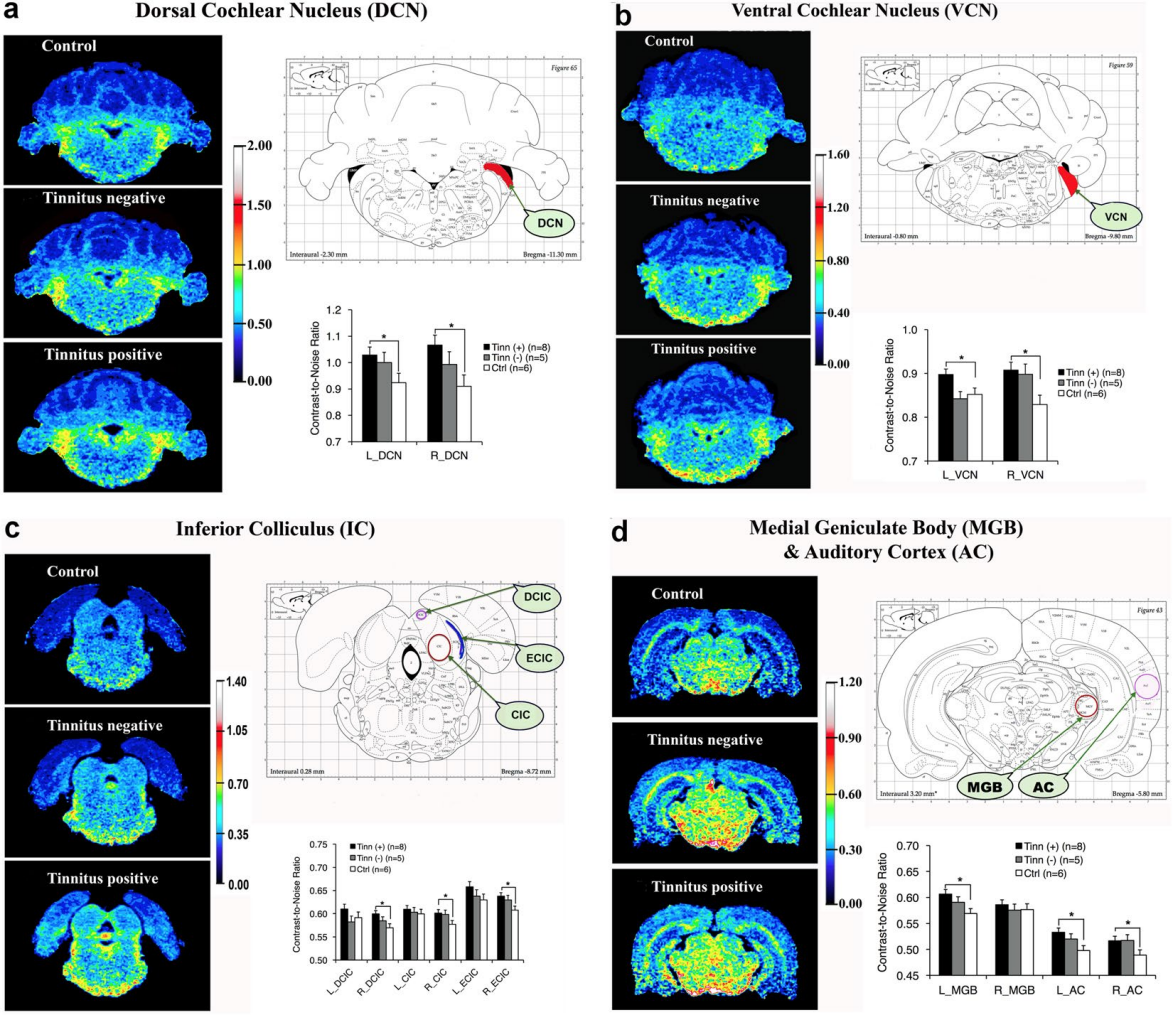
Blast-induced tinnitus (+) rats had higher neural activity in several central auditory structures compared to control (ctrl) rats, though there were no significant differences between tinnitus (+) and tinnitus (−) rats. Images were color-coded according to manganese uptake intensity, as defined by the color scale-bar. Color-coded images are shown for a representative tinnitus(+), tinnitus(−) and control (ctrl) rat. A rat brain atlas was used to guide ROI placement. The bar graphs indicate the averaged uptake values per group and brain region. (**a**) Significantly higher manganese uptake was found in the left and right DCNs of the tinnitus(+) group, compared to the control group, as well as in (**b**) the left and right VCNs; (**c**) the right DCIC, CIC and ECIC, and; (**d**) the left MGB and the left and right ACs. For all graphs, error bars represent standard error of the mean. *Indicates statistical significance (p < 0.05). Atlases were adapted from The Rat Brain In Sterotaxic Coordinates, 4^th^ Edition by Paxinos and Watson (1998) with permission from the publisher.

There were significant overall group differences in manganese uptake in the auditory structures ipsilateral (*F*_(2,16)_ = 5.073, *p* = 0.020) and contralateral (*F*_(2,16)_ = 6.590, *p* = 0.008) to blast exposure. Bonferroni adjustments revealed that the tinnitus(+) group showed higher manganese uptake compared to controls in the auditory structures that were either ipsilateral (*p* = 0.017) or contralateral (*p* = 0.007) to blast exposure. Among individual structures (Fig. 3a–d), post-hoc t-tests showed higher manganese uptake in tinnitus(+) rats compared to controls in the left and right DCNs (left *t*[12] = 3.005 *p* = 0.011; right *t*[12] = 2.668 *p* = 0.021; Fig. 3a) and VCNs (left *t*[12] = 2.281 *p* = 0.042; right *t*[12] = 3.387 *p* = 0.005; Fig. 3b), the right DCIC (*t*[12] = 3.426 *p* = 0.005), right CIC (*t*[12] = 2.330 *p* = 0.038), and right ECIC (*t*[12] = 3.558 *p* = 0.004; Fig. 3c), the left medial geniculate body (MGB) (*t*[12] = 2.668 *p* = 0.020; Fig. 3d), and the left and right ACs (left *t*[12] = 2.923 *p* = 0.013; right *t*[12] = 2.428 *p* = 0.032; Fig. 3d). Overall, these findings suggest that while blast-induced tinnitus may play a role in the enhanced auditory neural activity, other effects of blast impact and potentially-induced TBI must also be taken into account.

Lastly, we compared rats that were blast-exposed under ketamine/xylazine anesthesia with those exposed under isoflurane and found no significant differences between groups (*F*_(1,11)_ = 1.946, *p* = 0.190) or any interactions between group and brain region (*F*_(1.343,6)_ = 2.285, *p* = 0.147). This suggests that anesthetization alone did not significantly influence manganese uptake in the auditory brain structures.

### Manganese-enhanced MRI (MEMRI) – limbic structures

**Figure 4:**
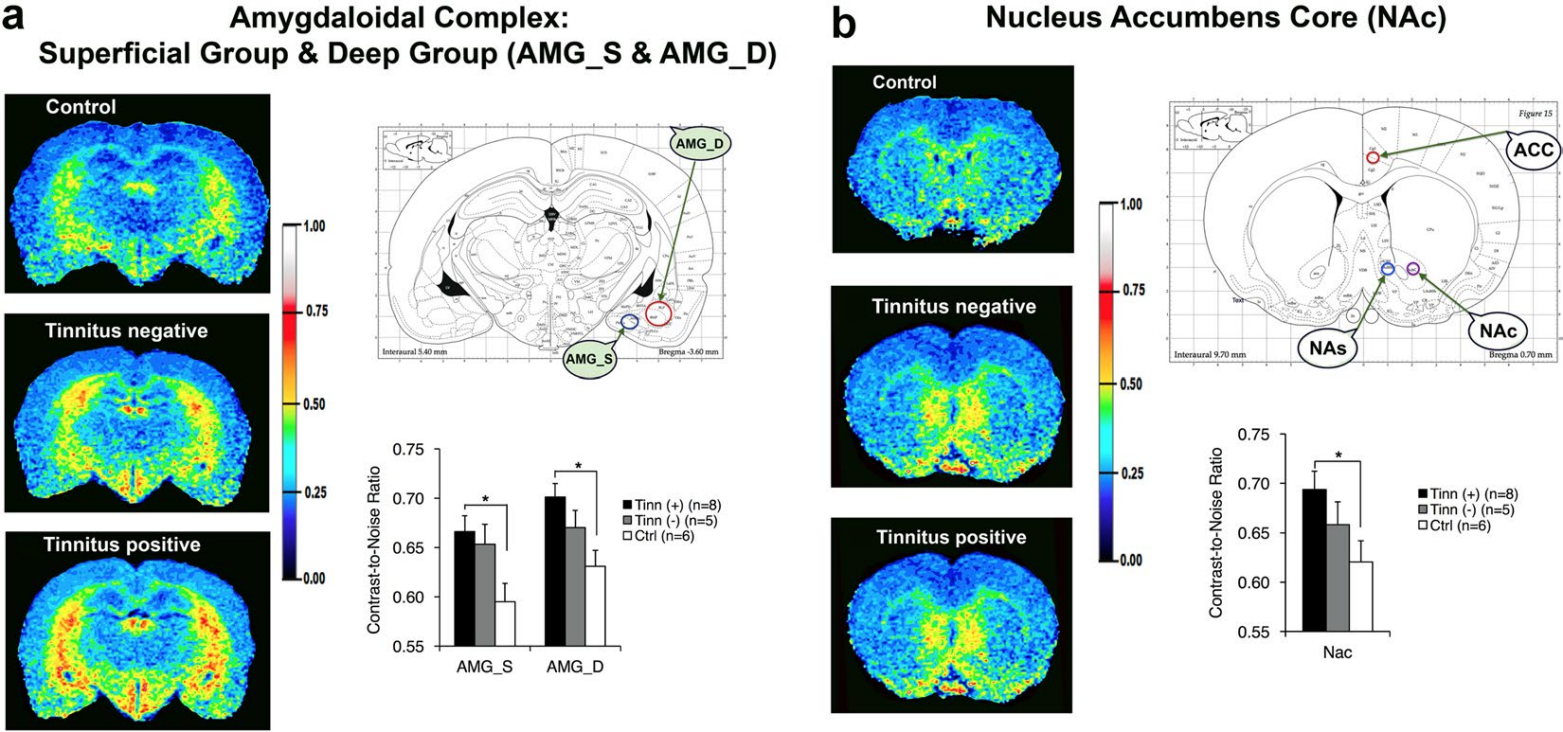
Blast-induced tinnitus (+) rats had higher neural activity in two amygdala subdivisions and in the nucleus accumbens core (NAc) compared to control (ctrl) rats, though there were no significant differences between tinnitus (+) and tinnitus (−) rats. Images were color-coded according to manganese uptake intensity, as defined by the color scale-bar. Color-coded images are shown for a representative tinnitus(+), tinnitus(−) and control (ctrl) rat. A rat brain atlas was used to guide ROI placement. The bar graphs indicate the averaged uptake values per group and brain region. (**a**) Significantly higher manganese uptake was observed in the superficial and deep amygdalae (AMG_S_ and AMG_D_) of the tinnitus(+) group, compared to the control group, as well as in the (**b**) nucleus accumbens core regions (NA_C_). *Indicates statistical significance (p < 0.05). Atlases were adapted from The Rat Brain In Sterotaxic Coordinates, 4^th^ Edition by Paxinos and Watson (1998) with permission from the publisher.

MEMRI was also conducted to determine whether blasted rats with or without tinnitus experienced changed neural activity in the limbic system. Repeated measures ANOVA revealed an overall group effect (*F*_(2,16)_ = 4.555, *p* = 0.027) when comparing the three groups, with the tinnitus(+) group exhibiting higher contrast-to-noise ratios (CNRs) in the limbic structures compared to the control group (Bonferroni adjustment *p* = 0.025; Table 2). Specifically, compared to the control group, post-hoc t-tests showed that the tinnitus group had greater manganese accumulation in the superficial/cortical-like amygdala (AMG_S_; *t*[12] = 2.963 *p* = 0.012; Fig. 4a), the deep/basolateral amygdala (AMG_D_; *t*[12] = 3.367 *p* = 0.006; Fig. 4a), and the nucleus accumbens core (NA_C_; *t*[12] = 2.374 *p* = 0.035; Fig. 4b). This indicated that blast-induced tinnitus may also play a role in the hyperactivity in the limbic system. No significant group differences or interactions between group and brain region, however, were observed between the tinnitus(+) and tinnitus(−) groups, or between the tinnitus(−) and control group (Bonferroni adjustment *p* = 0.315). This indicates that the non-auditory impact of blast and potentially-induced TBI may have also contributed to any observed limbic hyperactivity.

**Table 2:** Contrast-to-noise ratios for all limbic structures for the tinnitus(+), tinnitus(−), and control groups. Data are presented as mean ± standard error of the mean. *Indicates statistical significance compared to control group, according to post-hoc t-tests (p < 0.05).

MEMRI: Contrast-to-Noise Ratios			
Region	Tinnitus ( + ), (n = 8)	Tinnitus (−), (n = 6)	Control, (n = 5)
*AMG*_*S*_	*0.67 ± 0.02	0.65 ± 0.02	0.59 ± 0.02
*AMG*_*D*_	*0.70 ± 0.01	0.67 ± 0.02	0.63 ± 0.02
*AMG*_*C*_	0.67 ± 0.02	0.65 ± 0.02	0.63 ± 0.02
*NA*_*C*_	*0.69 ± 0.02	0.66 ± 0.02	0.63 ± 0.02
*NA*_*S*_	0.68 ± 0.02	0.64 ± 0.02	0.62 ± 0.02
*ACC*	0.48 ± 0.01	0.48 ± 0.01	0.47 ± 0.01
*HIPP*	0.62 ± 0.03	0.63 ± 0.04	0.56 ± 0.04

Since statistical power was potentially a limiting factor, especially when interpreting the lack of statistical significance between tinnitus (+) and tinnitus (-) rats across the limbic and auditory structures, we computed the detectable differences at 80% power (Supplementary Table S1). The detectable differences for comparison of tinnitus positive versus tinnitus negative rats ranged from 0.038 for the anterior cingulate cortex (ACC) to 0.225 for the right dorsal cochlear nucleus (R-DCN). However, none of the differences in contrast to noise ratios between the tinnitus positive and tinnitus negative rats (see Supplementary Table S2) reached the detectable difference threshold, further suggesting that there were not meaningful differences between these two groups.

When comparing rats that were blast-exposed under ketamine/xylazine anesthesia with those exposed under isoflurane, repeated measures ANOVA showed no significant differences between groups (*F*_(1,11)_ = 1.727, *p* = 0.216) or any interactions between group and brain region (*F*_(2,6)_ = 0.506, *p* = 0.609). This suggests that anesthetization alone did not significantly influence manganese uptake in the limbic structures.

## Discussion

### Blast-induced tinnitus and hearing loss

In the current study, we observed that lasting tinnitus behavior manifested in some but not all blast-exposed animals. Tinnitus behavior manifested across a range of spectral frequencies, but most robustly at 26–28 kHz. Together, these trends match previous reports on animal models of blast-^52, 53, 65^ and noise-induced tinnitus^66, 67^. These findings also reflect the clinical situation, in that tinnitus may not disappear over time following blast exposure^68^, and that blast exposure induces lasting tinnitus in some but not all individuals^8^. This comparability supports the clinical applicability in using animal models to investigate blast-induced tinnitus, as well as other effects and mechanisms of blast exposure.

One potential limitation is that our gap-detection ratio values (ranging from 0.6–0.8) were a little higher than those reported in some other studies^9, 66^, which could raise concerns about reduced sensitivity of tinnitus detection. Nevertheless, 62% of our blast-exposed rats exhibited tinnitus behavior, which is a similar tinnitus yield to intense noise-exposure studies with baseline ratios predominantly below 0.6 (56% tinnitus yield^69^; 66% tinnitus yield^66^). In another study with gap-detection ratio values between 0.6–0.8, we found that 44–67% of rats exhibited lasting tinnitus and expressed significant hyperactivity in the IC and AC compared to tinnitus(−) rats^54, 55^. This, and the fact that the current tinnitus (-) and control animals did not significantly change their gap-detection and PPI ratios over the 5 weeks tested, supports the sensitivity and validity of our behavioral testing for tinnitus. Our gap-detection findings were further validated via startle force amplitude analysis (Fig. S1). That is, while some animals exhibited an increase or decrease in startle amplitude over the experimental timeline, this was not restricted to the tinnitus (+) group. This suggests that changes in startle amplitude did not result in false tinnitus positive results.

In addition to tinnitus, our findings also agreed with previous studies in that ABR hearing thresholds can recover following blast exposure^49, 52, 53^, depending on the parameters of blast. These studies have shown that a single blast exposure in the range of 94–150 kilopascals may induce reversible hearing loss below 30 kHz. These results reflect human studies as well, where both transient and lasting blast-induced hearing loss have been observed^68, 70, 71^. Mechanistically, an animal model has shown that outer hair cell and spiral ganglion loss can underlie blast-induced auditory dysfunction, though most especially for permanent hearing threshold shifts^49^. The peripheral auditory mechanisms of temporary blast-induced threshold shifts remain less well-known, and may thus be a relevant area for future investigation.

Despite hearing threshold recovery, a reduction in ABR wave P1-N1 amplitudes remained in the current blast-exposed animals. This correlates with our previous blast study^53^, noise exposure studies conducted by others^64, 72–74^, and reports on human tinnitus patients^75, 76^. Nevertheless, P1-N1 amplitude reductions in patients without tinnitus have also been reported^75^, and in the current study, no differences in amplitude reduction were seen between tinnitus(+) and tinnitus(−) rats, replicating previous studies^53, 77^. In fact, noise-exposed guinea pigs with tinnitus have actually showed larger P1 and N1 amplitudes^78^. Given that different species, acoustic trauma parameters, and assessment time following exposure have been used, the disparities between studies can be challenging to interpret and warrant further examination.

### Effects of tinnitus and blast on anxiety

Significant anxiety levels on the EPM were only observed when tinnitus(+) and tinnitus(−) rats were combined and compared to controls. Therefore, a clear correlation cannot be made between tinnitus and anxiety in the current results﻿ using the current blast parameters. Clinically, however, the link between tinnitus and anxiety has been well-established^41, 43, 44^. Previous studies in rats, it should be noted, have found that noise-induced tinnitus was also not always associated with an overall increase in anxiety^66, 79^. This may suggest that rats do not necessarily experience tinnitus-related distress if tinnitus does not reach a severe and bothersome level or that they express that distress in a different manner. The latter scenario is supported by other studies that showed significantly altered behavior in tinnitus(+) rats in impulse control and social interaction^79, 80^. Therefore, testing these and additional behaviors such as grooming microstructure, sucrose consumption, sleeping, and others, may help clarify tinnitus-related distress in animals and bolster their clinical relevance. Furthermore, the reality that some humans (and thus, potentially animals) with tinnitus do not always experience anxiety^81–83^ must be considered. Screening for high anxiety rats within experimental and control groups could be helpful in that regard^66^.

The fact that blast-exposed rats as a whole showed significant anxiety-like behavior suggests a role for blast and blast-related effects in the development of anxiety. This correlates with clinical data, where blast-exposed survivors can develop anxiety disorders such as PTSD, or other forms of psychological distress^84–89^. Both blast-induced anxiety and fear have been demonstrated in animals across a variety of behavioral tests^90–96^. Despite the similarities between clinical and animal findings, however, it is important to remember that animals are typically anesthetized during blast exposure and do not experience the additional stressors faced by many blast-exposed survivors (i.e. prolonged battle stress, graphic violence, etc.). Therefore, it is possible that blast influences emotional processing in animals by affecting their physiology. First, blast can cause an array of physiological damage, including vision, auditory and motor defects^49, 51, 65, 94, 95, 97^, and sustaining such damage may yield an emotional impact on animals. Second, TBI can be induced by blast wave exposure^98–101^ and is itself a potential trigger for anxiety in both animals^102, 103^ and humans^104, 105^. The presence and effect of blast-induced TBI (from 14 PSI blast) in the current study is unknown since we did not assess animals for TBI. However, others have shown that blast exposure as mild as 3–11 PSI could scatter hyperchromatic and apoptotic neurons in the cerebral cortex^106^, induce glial activation^94^, and degenerate neurons in the dentate gyrus^107^. Blast-induced TBI and anxious behavior has also been simultaneously verified in an animal model^96^. Accordingly, it is plausible that the current, blasted rats mainly exhibited anxiety-like behavior in response to physiological changes, including TBI.

### Effects of tinnitus and blast impact on cognition

In the current study, rats with blast-induced tinnitus did not exhibit significant spatial learning or memory deficits during MWM testing. The MWM is a robust test for cognitive impairment due to its high reliability, its validity in measuring spatial cognition, and its applicability in both animals and humans^108, 109^. Nevertheless, other cognitive studies conducted on rats with noise-induced tinnitus also saw normal spatial cognition, as well as reaction time accuracy^66, 80, 110^, though one did find that impulsive control was impaired in rats with tinnitus behavior^49^. Tests of cognitive impairment in humans have found mixed results as well, with some people showing deficits in working memory and reaction time^111, 112^, while others have lacked deficiencies in reaction time and recall tasks^37, 113^. Given this, while animal studies may have the potential to elucidate the multifaceted relationship between tinnitus and cognitive impairment in humans, they should be utilized to reveal significant, clinically-relevant dysfunction. Since symptoms like cognitive-behavioral impairment are a key problem associated with tinnitus, demonstrating their presence in animal models may be critical to better understand the mechanisms of tinnitus and improve overall clinical relevance. Controlled animal studies may also help tease apart the cognitive-behavioral effects of tinnitus from comorbidities like depression and anxiety, which alone can cause cognitive impairment^114^.

In addition to tinnitus, the fact that blast exposure and non-tinnitus effects themselves did not impair spatial cognition must also be considered. Humans subjected to blast exposure can experience cognitive impairment such as memory loss and difficulty concentrating^115, 116^. A complicating factor, however, is that these and related impairment can be attributed to blast-associated TBI or PTSD^117, 118^, both of which may occur independently and require different therapeutic approaches for optimal treatment. Mixed effects have been observed in other animal studies, with one finding no effect of blast-induced TBI on spatial cognition^119^, and others finding significant impairment^91, 120^. Since animals are generally not conscious during blast exposure, it may be most likely that the physiological effects of blast, such as TBI, are responsible for impairment. Cognitive dysfunction may depend on the level of blast-induced TBI and/or anxiety. Conversely, some types of cognitive functioning may simply be less susceptible (at least in animals) to blast exposure, TBI and the other effects of blast.

### Effects of tinnitus and blast on central auditory hyperactivity

We found broad hyperactivity in both ipsilateral and contralateral auditory centers when comparing tinnitus(+) rats with controls. However, since no significant differences were found between tinnitus(+) and tinnitus(−) rats, the hyperactivity in tinnitus(+) rats may not have been exclusively due to tinnitus. In other MEMRI studies, rats with noise-induced tinnitus had hyperactivity in the DCN, VCN, paraflocculus, DCIC, and the IC^59, 61^, while rats with salicylate-induced tinnitus displayed activation in the auditory brainstem and midbrain^60, 62^. Nevertheless, these studies are also unable to explicitly separate the non-tinnitus effects of acoustic trauma, or salicylate, from tinnitus since experimental animals were not separated into tinnitus(+) and tinnitus(−) groups. Such analysis must be conducted in future studies so that the tinnitus-specific effects of MEMRI can be identified. It is possible that tinnitus(+) animals simply suffer from more noise-induced hearing damage than tinnitus(−) animals, which may only be unveiled by detailed histological analysis of the peripheral and central auditory systems. It should be noted, however, that MEMRI has now shown comparable auditory hyperactivity induced by several different tinnitus generators, and has yielded findings in line with the auditory hyperactivity observed in fMRI studies of tinnitus patients^17, 18^. Central auditory hyperactivity has also been strongly implicated as a neural mechanism of tinnitus by using electrophysiology, gene expression, and other methods^14, 18, 121–129^. In our previous blast exposure work, we found that the induced tinnitus was correlated with injury and compensatory plastic changes in the central auditory system^52^, as well as early hyperactivity in the DCN and IC and delayed hyperactivity in the AC^53–55^. Thus, the shared data trends among these animal and human studies suggest that tinnitus possibly played some role in the current results.

Although the current rats underwent unilateral blast exposure, hyperactivity was found along both ipsilateral and contralateral auditory pathways. This becomes part of the wider question of whether the hyperactivity was due to the auditory (loud noise) or non-auditory (shockwave-induced shearing impact) components of the blast exposure. Although this cannot be absolutely determined in the current study, bilateral hyperactivity has been observed in other MEMRI studies following unilateral noise exposure to induce tinnitus^60, 61^. Downregulation of inhibition, or upregulation of excitation, has been suggested to occur bilaterally in the brainstem following unilateral auditory insult^130^. The CIC also receives bilateral input from numerous auditory structures^131, 132^, and at the thalamic and cortical levels, bilateral ascending innervations become more prominent. This suggests that it is possible that the auditory component of blast led to bilateral hyperactivity.

With respect to non-auditory factors, blast and TBI are known to cause broad, wide-ranging injury to the brain, and this may nonspecifically impact the central auditory system. For example, one blast-induced TBI study using MEMRI has reported broad, significant signal enhancement across the brain, though this disappeared by 1 month post-blast^133^. Other MEMRI studies that investigated plain impact of TBI have found a temporarily increased uptake in the dentate gyrus at 1 month post-blast^134^, as well as increased uptake in the core and decreased uptake in the area surrounding the core^135^. Blast-exposed animals as a whole have sustained microstructural changes in the IC and MGB^52^ and astrogliosis in the MGB^51^, while neuronal loss has been observed in the thalamus following plain TBI impact^136–138^. It is thus feasible that blast-induced TBI impact itself affects the central auditory system, or that it adds to the auditory component of blast-induced injury. The latter may be supported by the fact that humans with comorbid TBI have a higher propensity for tinnitus^9^. Clearly, further studies are needed to distinguish tinnitus- and auditory-specific effects from blast-induced TBI impact on the central auditory system. Longer post-blast assessment periods, for instance, could be implemented, since central auditory hyperactivity may sometimes take up to 3 months to consolidate^55^.

### Effects of tinnitus and blast on limbic hyperactivity

Tinnitus(+) rats exhibited a broad increase in manganese-related neural activity of two subdivisions of the amygdala (AMG_S_ and AMG_D_), and of the NA_C_, when compared to control rats. As with the central auditory system, no significant differences were found between tinnitus(+) and tinnitus(−) rats, and consequently, these findings may not be solely due to tinnitus. The fact that blasted animals as a whole displayed increased anxiety behavior compared to controls suggests that blast and potentially TBI may have also played a role in limbic activation.

Concerning tinnitus and auditory factors, two other MEMRI studies have evaluated tinnitus-related activity in the amygdala. One found that acute noise- and salicylate-induced tinnitus had no effect^60^, while another showed that rats with chronic noise-induced tinnitus actually had lower activity^59, 61^. A distinction in the current study is that we separately analyzed the amygdala subdivisions, which may have better enabled detection of activity changes. Other animal studies have shown that tinnitus inducers like noise exposure and salicylate injections can enhance Fos-like immunoreactivity^139, 140^, Arc gene expression^141^, as well as hyperactivity and tonotopic shifts^142, 143^ in the amygdala. Varied results have been found in human studies, with increased amygdalar blood flow noted in some patients^25, 144^ whereas others lacked amygdalar hyperactivity when emotionally processing sound^145^. While the nucleus accumbens has not been as well-studied as the amygdala, it has shown increased connectivity with the auditory cortices^146^, as well as hyperactivity^46^ and grey matter decrease^48^. Since some individuals with tinnitus do not apparently suffer from it^81–83^ or experience psychological comorbidities, it may be that hyperactivity in some limbic regions reflects an adverse reaction to tinnitus. As mentioned above, behavioral tests that can detect tinnitus-related distress in animals are necessary to verify this.

Regarding the non-auditory effects of blast and potential TBI on limbic activation, it is interesting that tinnitus(+) rats showed hyperactivity of the AMG_S_, which largely receives and projects olfactory information^147^. Olfactory disruption has been correlated with TBI^148^ and could potentially be due to blast-induced TBI. More broadly, blast exposure and related TBI have induced activation and fear-related changes in the amygdala^90, 96^, as well as inflammation and neurochemical changes leading to apoptosis in the nucleus accumbens^149^. Therefore, it is possible that higher neural activity in the amygdala and nucleus accumbens primarily results from blast wave impact. The fact that we did not observe significant hyperactivity in the limbic or central auditory systems of tinnitus(−) rats either further suggests that tinnitus plays some role in hyperactivity, or that tinnitus(+) rats may have sustained more of the non-auditory effects of blast, such as TBI. Overall, the findings from this study can be advanced by future work, which could include detailed peripheral and central histology, differing the intensity and number of blast exposures, and distinguishing blasted animals that experience cognitive-emotional impairment from those that do not.

## Materials and Methods

### Animal subjects

Thirty male Sprague-Dawley rats (60–70 days old at the beginning of experiments) were purchased from Harlan Laboratories and kept on a 12/12 hour light/dark cycle inside a federally approved animal vivarium. Eleven rats were excluded due to poor baseline gap detection and prepulse inhibition (PPI) performance or abnormal baseline auditory brainstem response (ABR) thresholds. Of the 19 remaining rats, 13 were blast-exposed while 6 served as age-matched controls. All experimental protocols and amendments were approved by the Institutional Animal Care and Use Committee at Wayne State University, and all procedures were conducted in accordance with the US federal animal research guidelines.

### Gap-detection for tinnitus tests and PPI for hearing detection tests

Gap-detection and PPI testing were conducted before (3 times/week) and after (2 times/week) blast exposure to measure tinnitus behavior and hearing detection, as described elsewhere^52, 53, 65–67, 150^. Briefly, a rat was placed in a custom-made restrainer and locked onto a platform inside a sound-attenuation chamber (Kinder Scientific, Poway, CA). Peak-to-baseline startle force of the rat was registered (in Newtons) using a piezoelectric transducer fixed underneath the platform.

For the gap detection procedure, background noise consisted of 2 kHz bandpass signals from 6–8, 10–12, 14–16, 18–20, or 26–28 kHz, or broadband noise (2–30 kHz), and delivered at 60 dB SPL. The startle stimulus consisted of a 50 ms noise burst delivered at 115 dB SPL. A 40 ms silent period beginning at 90 ms before the startle stimulus served as the gap. Startle force was recorded in response to 3 conditions: 1) background noise alone, 2) the startle stimulus preceded by the silent gap, or 3) the startle stimulus alone. For the PPI procedure, no background noise was administered. Instead, startle force was recorded in response to 1) the startle stimulus alone or, 2) the startle stimulus preceded by a 40 ms, 60 dB SPL acoustic prepulse beginning at 90 ms before the startle stimulus. Acoustic prepulses consisted of the same bandpass signals used for background noise in the gap-detection procedure.

### ABR recordings

Prior to blast exposure and at 5 weeks following blast, ABR testing was conducted to assess hearing thresholds. A rat was initially anesthetized with an isoflurane/air mixture (5% v/v and 1 L/min), which was reduced to 2% isoflurane/air for maintenance. The rat was placed on a warming blanket to sustain body temperature and secured to a stereotaxic frame. Three platinum-coated tungsten electrodes were inserted in the vertex, below the ipsilateral pinna, and in the contralateral temporal muscle for the active, return, and ground positions, respectively. Click and tone-burst stimuli at 8, 12, 16, 20, or 28 kHz were delivered from an electrostatic speaker through a tube inserted in the ear canal. Stimuli were presented in 5 dB SPL decrements ranging from 100 to 5 dB. The stimuli were generated by an RX6 multifunction processor and programmed by SigGenRP software (Tucker Davis Technologies System 3, Alachua FL). ABR signals were amplified, band-filtered from 0.3 to 3 kHz, notch-filtered at 60 Hz, and averaged 300 to 400 times for click and tone-burst stimuli, respectively.

### Blast procedure

A single blast exposure to induce tinnitus was conducted using a custom-made shock tube (ORA, Inc) located in the Wayne State University Bioengineering department. To avoid excessive trauma, each rat was placed on a platform 503 cm downstream from the bursting membrane and 112 cm upstream from the open end of the driven cylinder^151^. After anesthesia with either 4% isoflurane and oxygen (1 L/min) or a ketamine and xylazine mixture (100 mg/kg + 10 mg/kg IP), the right ear of each rat was plugged with Mack’s^®^ silicone putty and sealed with mineral oil. The rat was wrapped in a protective garment and secured on a holder in the driven cylinder. Peak static overpressure of 14 psi (96.5 kPa; 194 dB SPL) was produced with compressed helium and calibrated Mylar sheets (GE Richards Graphics Supplies Inc., Landsville, PA), the latter of which were placed between the driver and driven cylinder. Blast overpressure burst the Mylar membranes and generated a free-field blast wave similar to that produced by an explosive device^151^. Following blast exposure, each rat was carefully monitored until it regained consciousness.

### EPM test

Anxiety levels were measured at 5 weeks post-blast (two days after the last week 5 gap-detection test) with a 5-min trial on the elevated plus maze (EPM). The plus maze apparatus was a commercial model (Coulbourn Instruments, Allentown, PA) constructed from black Plexiglass. The apparatus has been described in detail elsewhere^152^. Rodent movement on the maze was recorded by a camcorder mounted on the ceiling and was analyzed with Ethovision XT version 6 software (Noldus Information Technology, Leesburg VA). A rat was placed in the center of the maze to initiate a trial.

### MWM test

At 5 weeks post-blast (2 days following EPM testing), spatial learning and memory were assessed using an one-day MWM approach, which has been validated by our lab and others^66, 107, 153, 154^. An one-day protocol was preferred to a longer protocol since tinnitus could potentially fluctuate during longer testing periods and simultaneous testing of rats for spatial cognition and the abovementioned tests would be stressful. To test rats, a circular fiberglass pool (183 cm diameter) was filled with water and opacified with black tempera paint. A hidden escape platform (11 cm diameter) was submerged 2 cm below the water surface level. Rats learned to use extra-maze cues to find the hidden platform, which was located in the target zone (quadrant 4) of the tank. Swimming trajectories of each rat were recorded using a digital camcorder mounted on the ceiling and analyzed with Ethovision software. Escape latency and time spent in the target zone were calculated.

### Escape latency trials

Each rat swam a total of twelve trials, which were divided into three blocks with four trials apiece. The rat was gently lowered into the water facing the pool wall at one of four random starting positions (N, S, E, W). If the rat failed to locate the hidden platform within 60 seconds, it was manually placed onto the platform for an association period of 3 seconds. Thirty-minute breaks were given between blocks.

### Probe trial

Thirty minutes following the last escape latency trial, rats were tested with one probe trial. Here, the platform was removed and a rat was allowed to swim in the pool for 60 seconds.

### MEMRI scanning

At 5 weeks following blast, imaging to assess central auditory and limbic activity changes was conducted using a 7.0 T Siemens ClinScan MRI scanner (Siemens Medical Solutions USA, Inc. Malvern, PA). Rats were injected with MnCl_2_ (67 mg/kg body weight) intraperitoneally and were placed in their home cages in a soundproof room for 8 hours to allow uptake of manganese. They were occasionally monitored through an observation window for signs of distress (i.e. coat appearance, unusual mobility), though no signs of distress were observed. Before scanning, a rat was anesthetized with a 4% isoflurane/air mixture in an induction chamber. Anesthesia was subsequently maintained with 2% isoflurane/air via a commercially made MRI compatible nose cone. During scanning, the rat was placed on a heated re-circulating water pad to maintain core body temperature. A whole-body transmit-only coil and a 4-element Bruker mouse-brain receive-only surface coil placed dorsal to the rat’s head were used for scanning. T1-weighted and 3D gradient-echo images were acquired. Individual images were acquired with 2 sets of 3-D Turbo-Flash sequence (repetition time: 7.7 ms, inversion time: 1500 ms, echo time: 3 ms, flip angle: 3°, 192 × 192 × 128 matrix, 2.50 × 2.50 × 3.32 cm field of view). The magnetization-prepared rapid acquisition gradient-echo (MPRAGE) image sequences were acquired with inversion pulses while the proton density–weighted gradient-echo (PDGE) image sequences were acquired without inversion pulses. The thickness of the slice between adjacent images was 0.26 mm.

### Data Analyses

#### Gap-detection/PPI for tinnitus evaluation

To determine tinnitus presence, gap-detection data were divided into ratios as previously described^52, 53, 65–67, 150^. Values close to 0 would indicate strong suppression of the startle reflex in response to silent gaps, and thus healthy status, whereas a value close to 1 would signify little suppression in response to the gap, potentially indicating tinnitus^52, 67, 150, 155–160^. PPI data were analyzed in the same manner, and lower or higher PPI ratio values would indicate healthy or compromised hearing detection, respectively. Ten of the original 30 rats were excluded from the study due to an inability to generate significant suppression of the acoustic startle reflex in response to silent gaps or acoustic prepulses, compared to trials with only the startle stimulus. A rat was considered tinnitus(+) if it exhibited a significantly elevated post-blast gap-detection ratio (compared to pre-blast) and was no longer able to significantly suppress its startle reflex in response to the silent gap, as detailed elsewhere^66^. Rats that exhibited no elevations in post-blast gap-detection ratios were considered tinnitus(−). Control rats were assessed similarly to ensure that they did not spontaneously develop tinnitus. To expand on individual analysis, data were also examined at the group-level (tinnitus(+), tinnitus(−), or control). If post-blast ratios were significantly higher than pre-blast ratios, this would indicate tinnitus and/or hearing detection loss, respectively, for that group as a whole. Startle amplitude of the startle only responses were also assessed, since a decrease in startle amplitude could result in false tinnitus-positive behavior^63^. Thus, post-blast startle amplitude, relative to pre-blast startle amplitude, was determined for each animal.

Lastly, we compared gap-detection ratios between rats blasted under ketamine/xylazine with those blasted under isoflurane. This analysis was conducted to determine whether anesthesia later affected tinnitus development.

#### ABR data

ABR thresholds and wave P1-N1 amplitudes were measured to further assess hearing loss and were compared between groups. Thresholds were considered to be the lowest sound intensity at which a distinct portion of the biological ABR waveform remained visible. Higher ABR thresholds relative to other groups would indicate hearing loss. One rat from the original 30 was excluded from the study due to baseline ABR thresholds far outside of the normal range (its thresholds were 60 dB SPL or greater). Since neuropathology can exist in spite of ABR threshold recovery^64^, especially at high frequencies^76^, we measured wave P1-N1 amplitude in response to 28 kHz tone-bursts at 5 weeks post-blast. The 28 kHz was chosen because at this frequency, behavioral evidence of tinnitus was the most robust. Smaller amplitudes relative to the control group would indicate cochlear nerve damage or ribbon synapse loss^64^, and some have suggested that P1-N1 degradation can influence gap-detection^72^. Finally, we compared hearing thresholds and P1-N1 amplitude between animals blasted under ketamine/xylazine with those blasted under isoflurane.

#### EPM data

Anxiety-like behavior was assessed by calculating the percentage of entries into and time spent in the open-arms. Reduced entries and time in the open-arm would indicate higher anxiety, while increased entries and time would suggest less anxiety. The raw number of open-arm and closed-arm entries were also compared between groups to account for potential differences in mobility, which could occur following blast exposure.

#### MWM data

Data were compared between groups to assess spatial learning and memory. Spatial learning was evaluated by the escape latency trials while spatial memory was gauged in the probe trial by the amount of time spent in the target zone. Longer escape latencies and a lower affinity for the target zone would suggest impaired spatial learning and memory.

#### MEMRI data

**Figure 5:**
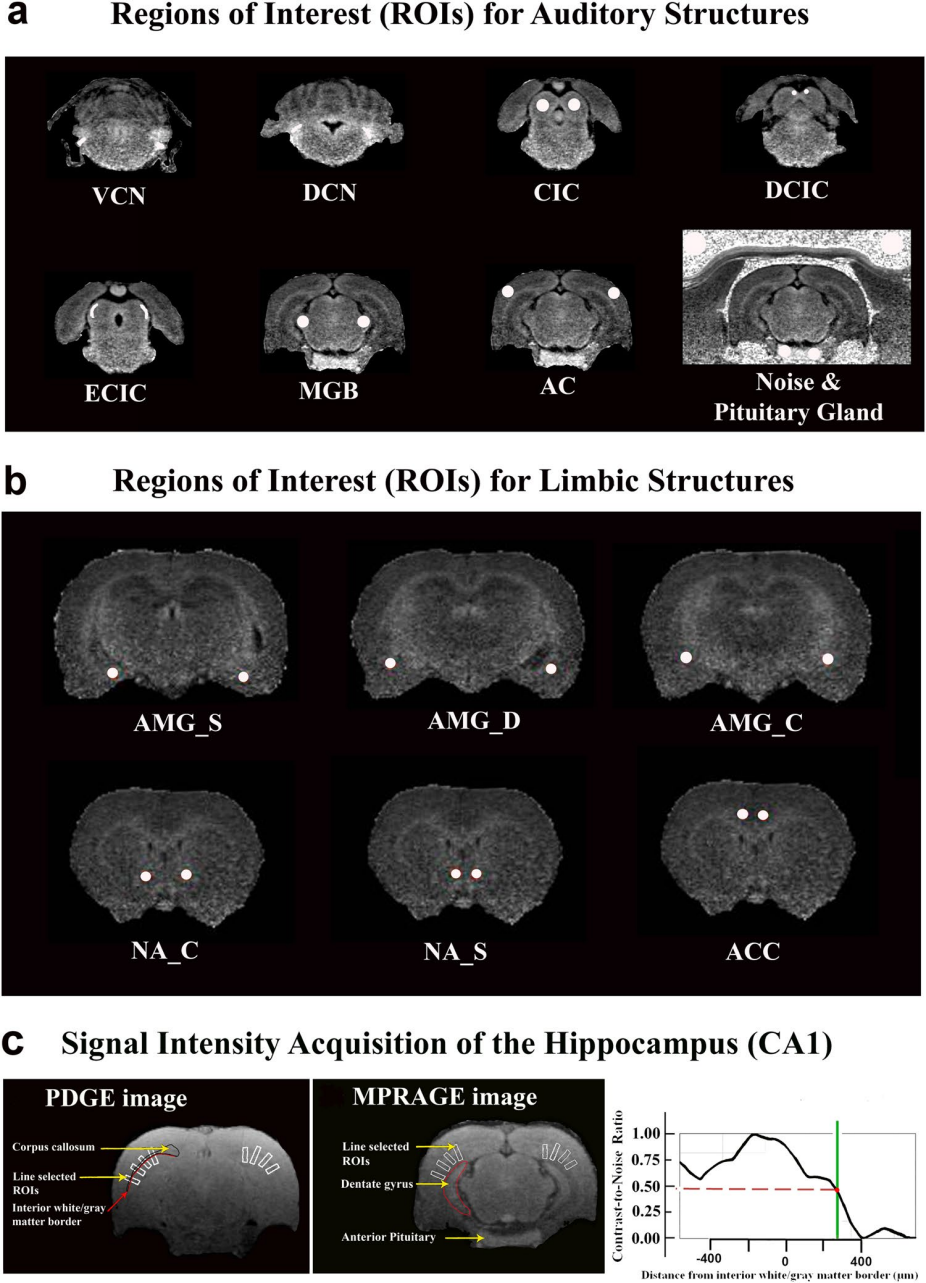
Representative ROI placements. (**a**) To measure manganese uptake in the auditory system, ROIs were placed in the left and right dorsal cochlear nuclei (DCNs), ventral cochlear nuclei (VCNs), central nuclei of the inferior colliculus (CICs), external cortices of the inferior colliculus (ECICs), dorsal cortices of the inferior colliculus (DCIC), medial geniculate bodies (MGBs), and auditory cortices (ACs). ROIs were also placed in the left and right anterior pituitary glands and nearby noise. (**b**) To measure the limbic system, ROIs were placed in the left and right centromedial amygdala (AMG_C_), the superficial/cortical-like amygdala (AMG_S_), deep/basolateral amygdala complex (AMG_D_), nucleus accumbens core (NA_C_) and shell (NA_S_), and anterior cingulate cortices (ACCs). (**c**) To measure the hippocampus, four rectangular bands (white color) were first placed onto the PDGE image. They were then copied onto the corresponding MPRAGE image.

Images were processed with in-house R scripts (v2.12.1, http://www.r-project.org). MPRAGE image sequences were divided by PDGE sequences to create heavily T1-weighted ratio values^161, 162^. The corrected images were then used for analysis. Regions of interest (ROIs) were placed to generate signal intensities and measure manganese uptake using MRIcro v.1.40 software (Fig. 5a–c), with the Paxinos and Watson rat brain atlas^163^ used as a reference. For the central auditory system, we analyzed the dorsal cochlear nucleus (DCN), ventral cochlear nucleus (VCN), central nucleus of the inferior colliculus (CIC), external and dorsal cortices of the inferior colliculus (ECIC and DCIC), medial geniculate body (MGB), and the auditory cortex (AC). For the limbic system, we analyzed the amygdala via three subdivisions (the centromedial nuclei, AMG_C_; the superficial/cortical-like nuclei, AMG_S_; and the deep/basolateral nuclei complex AMG_D_^147^), as well as the anterior cingulate cortex (ACC), the nucleus accumbens core (NA_C_) and shell (NA_S_), and the hippocampus (HIPP). The anterior pituitary was also analyzed to determine whether non-specific, systemic brain enhancement occurred^164^.

ROIs were manually drawn in the DCN and VCN, spanning 2 and 3 consecutive coronal slices, respectively. Spherical 3D ROIs were used to characterize the IC, MGB, and AC (diameter: 520 microns). ROIs were placed at the rostrocaudal center of each anatomical region and drawn to occupy the entire coronal profile of each region, excluding a buffer ( ≥ 1 voxel wide) at the borders with neighboring brain regions and tissues. Spherical 3D ROIs were also used to characterize the amygdala subdivisions, the ACC, and the NA_C_ and NA_S_. The AMG_C_ ROIs were placed so that they were immediately rostral to the lateral ventricles. The AMG_S_ ROI was placed immediately lateral and ventral to the lateral ventricles, while the AMG_D_ ROI was placed lateral, dorsal, and rostral to the lateral and 4^th^ ventricles. Nucleus accumbens ROIs were placed so that the optic nerves were visible. The NA_C_ ROI was placed lateral and ventral to the lateral ventricles, while the NA_S_ ROI was placed medial and ventral to the lateral ventricles. ACC ROIs were placed so that the optic nerves were visible and the ROIs were dorsal and medial to the corpus callosum. ROI placement was confirmed using the parasagittal and transverse profile views. Two blinded raters independently placed ROIs and their values were subsequently averaged. The averaged ROI signal intensities were then normalized to that of adjacent noise to produce contrast to noise ratios (CNR = SI__ROI_/SI__noise_), which have been used by others^165, 166^.

For the HIPP, signal intensities were collected in conjunction with ImageJ and based on a previous approach^162^. Three consecutive slices were used for each hemisphere. The first slice was caudal to the slice where the dentate gyrus became evident. Four rectangular bands were drawn on each side so that 1) they were perpendicular to the curvature of the forceps major of the corpus callosum; 2) the midpoint of the band aligned with the border of the forceps major, and; 3) the direction of the band was toward the center of the parenchyma (Fig. 5c). The bands were drawn on the PDGE image and then copied to the MPRAGE image on the same corresponding coronal slice. The MGB was clearly visible on slices where the bands were placed.

Higher contrast-to-noise ratios (CNRs) in a given group compared to the other groups (i.e. tinnitus(+) *vs*. control) would indicate greater, general activity in that respective brain region. Higher CNRs in the auditory pathway ipsilateral to blast exposure versus the contralateral pathway would demonstrate a lateral effect. Manganese uptake in rats blast-exposed under ketamine and xylazine anesthesia was also compared with that of rats exposed under isoflurane to determine if anesthesia was a confounding factor.

#### Statistics

Repeated measures ANOVAs were used to assess gap-detection, PPI, ABR, MEMRI, and MWM data. Greenhouse-Geisser corrections were used when Mauchly’s test of sphericity was violated. Significant results were followed by post-hoc t-tests where appropriate. All p values reported have been corrected with Bonferroni adjustments when multiple comparisons were conducted. EPM data, and MEMRI CNR data from the anterior pituitary and noise were assessed with one-way ANOVA. *P* < 0.05 was considered significant. Statistical analysis was performed with IBM SPSS 21.0 software (Chicago, IL).

Since small sample sizes were a potential limiting factor for the MEMRI data, the detectable differences between the groups for the 14 auditory structures and 7 limbic structures were calculated. We first performed a calculation for alpha = 0.05 (with no Bonferroni adjustment) and then a calculation for alpha = 0.017 (with Bonferroni adjustment for three comparisons). The pooled variances and pooled standard deviations were computed next. Finally, the general detectable effect sizes were computed assuming SD = 1 (using nQuery Advisor), and then the pooled standard deviation estimates were computed to obtain the MEMRI specific differences detectable with 80% power.

## Electronic supplementary material

Supplementary Information
