# Supplementary material for: Blast-Induced Tinnitus and Elevated Central Auditory and Limbic Activity in Rats: A Manganese-Enhanced MRI and Behavioral Study
Source: Sci Rep. 2017 Jul 7;7:4852. doi: 10.1038/s41598-017-04941-w (PMC5501813; doi:10.1038/s41598-017-04941-w)
Supplement: Supplementary file 1 — Supplementary Information [file 41598_2017_4941_MOESM1_ESM.pdf]

Supplementary Material

Blast-Induced Tinnitus and Elevated Central Auditory and Limbic Activity in Rats: A Manganese-Enhanced MRI and Behavioral Study

Jessica Ouyang(1) †, Edward Pace(1) †, Laura Lepczyk(1) †, Michael Kaufman(1), Jessica Zhang(1), Shane A. Perrine(2), Jinsheng Zhang(1)(3)*

[1] *Department of Otolaryngology and Head and Neck Surgery, Wayne State University School of Medicine, Detroit, MI, USA*

[2] *Department of Psychiatry and Behavioral Neurosciences, Wayne State University School of Medicine, Detroit, MI, USA*

[3] *Department of Communication Sciences & Disorders, Wayne State University College of Liberal Arts and Sciences, Detroit, MI, USA*

Supplementary Table S1: Effect size and detectable differences calculated between groups, including before and after Bonferroni corrections for multiple comparisons. Of note, the differences between the contrast-to-noise ratios of tinnitus positive and tinnitus negative rats (see Supplementary Table S2) did not exceed the detectable differences listed here in Table S1.

MEMRI: Detectable Differences

**Variable**	**T+ (n=8) vs. T- (n=5)**		**T+ (n=8) vs. Ctrl (n=6)**		**T- (n=5) vs. Ctrl (n=6)**	
	p = 0.05	p = 0.017	p = 0.05	p = 0.017	p = 0.05	p = 0.017
Effect Size	1.755	2.122	1.649	1.985	1.907	2.335
L-DCN	0.154	0.187	0.145	0.175	0.168	0.205
L-VCN	0.063	0.076	0.059	0.071	0.068	0.083
L-DCIC	0.050	0.060	0.047	0.056	0.054	0.066
L-CIC	0.041	0.049	0.038	0.046	0.044	0.054
L-ECIC	0.055	0.067	0.052	0.062	0.060	0.073
L-MGB	0.041	0.050	0.039	0.047	0.045	0.055
L-AC	0.040	0.048	0.038	0.045	0.044	0.053
R-DCN	0.186	0.225	0.175	0.210	0.202	0.247
R-VCN	0.092	0.111	0.086	0.104	0.100	0.122
R-DCIC	0.035	0.042	0.033	0.040	0.038	0.047
R-CIC	0.035	0.043	0.033	0.040	0.038	0.047
R-ECIC	0.039	0.047	0.037	0.044	0.042	0.052
R-MGB	0.047	0.057	0.044	0.053	0.051	0.063
R-AC	0.044	0.053	0.041	0.050	0.048	0.058
AMG_S	0.080	0.097	0.075	0.091	0.087	0.107
AMG_D	0.069	0.083	0.064	0.078	0.075	0.091
AMG_C	0.081	0.098	0.076	0.092	0.088	0.108

NA_C	0.090	0.109	0.085	0.102	0.098	0.120
NA_S	0.091	0.109	0.085	0.102	0.098	0.120
ACC	0.031	0.038	0.030	0.036	0.034	0.042
$HIPP$	0.157	0.190	0.148	0.178	0.171	0.209

Supplementary Table S2: Contrast-to-noise ratio difference between the three groups.

MEMRI: Contrast-to-Noise Ratio Differences

Region	T+ (n=8) vs. T- (n=5)	T+ (n=8) vs. Ctrl (n=6)	T- (n=5) vs. Ctrl (n=6)
L-DCN	0.028	0.104	0.076
L-VCN	0.055	0.045	-0.010
L-DCIC	0.028	0.019	-0.009
L-CIC	0.006	0.009	0.003
L-ECIC	0.020	0.028	0.008
L-MGB	0.016	0.038	0.022
L-AC	0.013	0.035	0.022
R-DCN	0.073	0.157	0.083
R-VCN	0.009	0.078	0.069
R-DCIC	0.015	0.030	0.015
R-CIC	0.003	0.024	0.021
R-ECIC	0.015	0.037	0.022
R-MGB	0.011	0.009	-0.001
R-AC	0.000	0.028	0.028
AMG_S	0.013	0.071	0.058
AMG_D	0.031	0.070	0.039
AMG_C	0.020	0.041	0.021
NA_C	0.044	0.049	0.005
NA_S	0.036	0.073	0.038
ACC	-0.002	0.014	0.016

HIPP	-0.004	0.065	0.069

Supplementary Figure S1

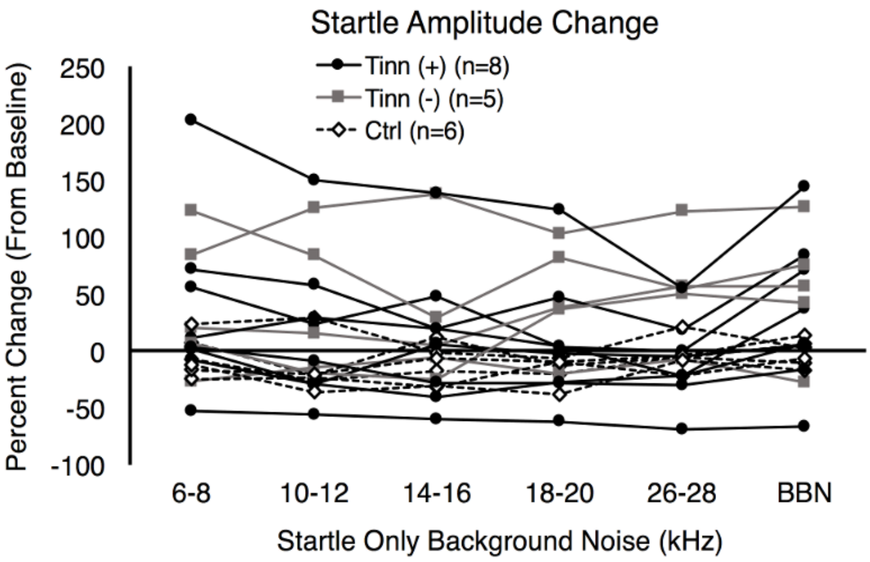

Figure S1: Percent change in startle amplitude to the Startle Only condition (of gap-detection test) in individual animals. Relative to baseline, startle amplitude either remained the same, increased, or decreased in individual animals after blast or sham-blast. However, these changes are not strictly observed in tinnitus positive animals, suggesting that startle amplitude did not confound tinnitus diagnosis.

Supplementary Figure S2

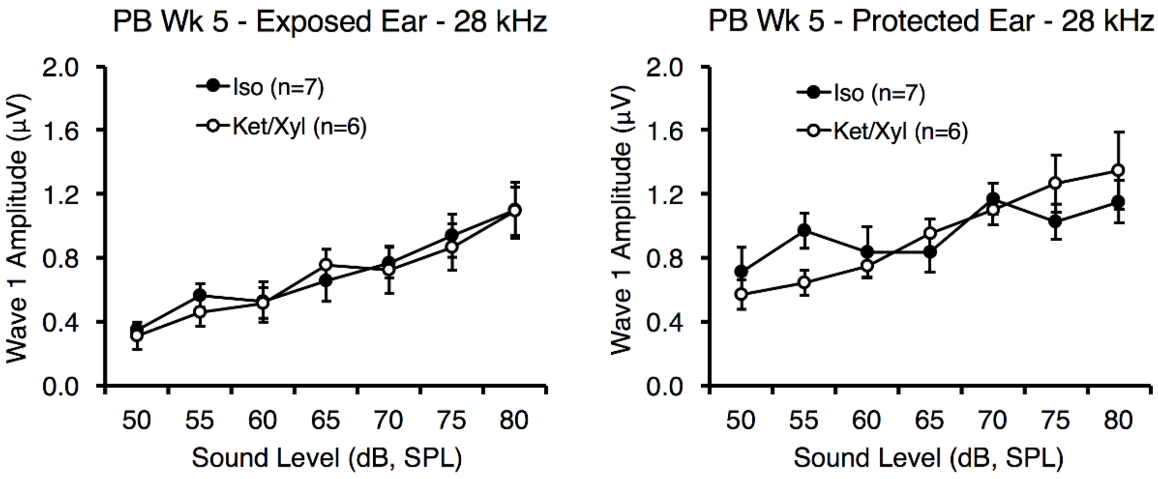

Figure S2: ABR P1-N1 amplitude in rats blasted under isoflurane (Iso) vs. rats blasted under ketamine/xylazine (Ket/Xyl). No significant differences were observed in P1-N1 amplitude between the two groups in either ear, suggesting a similar degree of blast-induced "hidden hearing loss".
